# Effect of Moisture Content of Fresh Pecans on Mechanical Shelling Efficiency, Nutritional Features, and Packaging Method

**DOI:** 10.3390/foods14050757

**Published:** 2025-02-23

**Authors:** Wenyue Tan, Yunfang He, Ziyan Wang, Yang Ni, Mengyang Xu, Jianke Du, Chenghang Liu, Xiaodong Jia

**Affiliations:** 1 Jiangsu Key Laboratory for the Research and Utilization of Plant Resources, Institute of Botany, Jiangsu Province and Chinese Academy of Sciences (Nanjing Botanical Garden, Mem. Sun Yat-Sen), Xuanwu District, Nanjing 210014, China; tanatanwy@163.com (W.T.); 15180074462@163.com (Y.H.); wangzizi1118@163.com (Z.W.); niyang93@163.com (Y.N.); xmy15366032805@163.com (M.X.); 15295595190@163.com (J.D.); chenghangliu@cnbg.net (C.L.); 2 Graduate School, Nanjing University of Chinese Medicine, Nanjing 210023, China

**Keywords:** *Carya illinoinensis*, fresh pecan, shelling efficiency, fruit quality, packaging method, storage

## Abstract

Fresh pecans are increasingly popular for their sweet taste and high nutritional value. To facilitate their commercialization, it is crucial to screen the proper moisture content for efficient mechanical shelling while retaining nutritional quality and finding a reasonable packaging method for storage. This study compared the mechanical shelling efficiencies of fresh pecans with different moisture contents via a standardized evaluation system used by the U.S. Department of Agriculture for over 70 years. The results indicated that pecans dried for 24 h (17.51% moisture, wet basis) achieved the highest mechanical shelling efficiency with the lowest kernel shoulders damaged (DSh%, 31.7%), shortest separation time (10.67 min·kg^−1^), and highest rate of complete halves (CH, 91.6%). Compared with dried pecans, fresh pecans had a lighter testa color (L*, 55.05), higher 2,2-Diphenyl-1-picrylhydrazyl (DPPH, 18.88 μg TE·g^−1^) and 2,2′-Azino-bis (3-ethylbenzothiazoline-6-sulphonic acid) diammonium salt (ABTS, 87.15 μmol TE·g^−1^), free-radical scavenging activity, and lower acid values (AV, 0.21 mg·g^−1^) and peroxide values (PV, 0.003 mg·g^−1^). Aluminum film packaging with vacuum (ALV) best preserved the quality of fresh pecans during 9 months of storage, as indicated by the acid and peroxide values. The results of this study provided a first report for the industrialization of fresh pecans.

## 1. Introduction

Pecan [*Carya illinoinensis* (Wangenh.) K. Koch], which belongs to the Juglandaceae family and *Carya* genus, is rich in nutrients such as lipids, phenolics, proteins, tocopherols, phytosterols, and minerals in its kernels [1,2]. Pecan kernels have been reported to have effects on lowering plasma lipids and improving the antioxidant status [3]. Pecan snacks are popular among the public, and the total consumption rises yearly. Utilized production in the U.S. totaled 271 million pounds in 2023 [4]. However, pecans are predominantly consumed as dried nuts, with moisture content maintained under 4% through hot air drying, sun drying, or shade drying to ensure a longer shelf life [5]. In addition, pecans are often roasted with additives such as sugar and salt to bring out their special flavor but also promote oil oxidation within the kernels and reduce the quality of pecans [6,7]. Furthermore, the evaluation of the freshness of pecan kernels becomes more difficult after additives are introduced. In recent years, the clear advantages of consuming fresh kernels over dried kernels have gradually been discovered, and the distinctive flavor of fresh kernels has gained popularity in several countries [8]. Fresh pecan kernels are non-astringent and sweet in flavor, making them suitable for fresh consumption. With less processing, fresh pecans preserve their original flavor, as well as more nutrients, aligning with consumers’ growing demand for fresh, convenient, and healthy foods. However, several issues remain to be addressed in the production and storage during the process of fresh pecan nuts to fresh pecan kernels.

Pecans are generally harvested mechanically by shaking the tree to dislodge the ripe nuts and then using a sweeper to collect the nuts from the ground [9]. After drying, the nuts are split into different-sized kernels through cracking, shelling, and blowing processes, the U.S. Department of Agriculture further graded the pecan kernels differently based on color and size [10]. At harvest, the moisture content of the fresh pecan kernels can exceed 30%, which is higher than traditional dried pecans, so it is crucial to determine the appropriate moisture content to preserve fresh taste, facilitate shelling, and extend the shelf life. For the commercialization of fresh pecans, modern production efficiencies must be considered from the outset, making it essential to compare mechanical shelling efficiencies. Complete pecans command the highest market value and retail price, while small pieces of kernels are sold at a lower price and are often used as an accessory in food processing. In addition, complete halves of pecans also have a longer shelf life than smaller pieces and are less susceptible to oxidation and other quality-degradation problems because of the smaller exposed surface area [11]. Thus, the percentage of complete halves is an important criterion for shelling efficiency. The traditional method of improving commercial shelling is to soak pecans with a moisture content of about 4% in hot water at varying temperatures or steam fumigate them for different periods before drying them again [12], but this method will cause cost and time waste and increase the possibility of the oxidation of pecan kernels. In this study, fresh pecans were dried for different lengths of time to directly reduce the moisture to a suitable moisture content for shelling, simplifying the rehydration and re-drying steps while saving time and production costs.

Excessive moisture content accelerates metabolism, making fresh kernels more perishable and shortening their shelf life [13], thus limiting the scope of the sale. Therefore, selecting an appropriate packaging method is essential. Current research on the preservation of fresh kernels has primarily focused on the application of edible coatings [14,15,16] and ultraviolet exposure [17], but these chemical and physical methods need to be accepted by consumers. Low-density polypropylene, polypropylene, polyethylene-nylon, and metal laminates have been applied to the storage of dried pecan kernels [18,19]. The combination of 83% N_2_ + 15% CO_2_ + 2% O_2_ effectively maintains the quality of fresh pistachios, while shelf life can be extended to about 3 months when stored at low temperatures [20]. Using an oxygen absorber in the package, raw, unpeeled almonds have a shelf life of at least 12 months, regardless of light and storage temperature [21]. The use of packaging materials with excellent barrier properties, or altering the atmosphere of the package, seems to be an appropriate way to package fresh pecans without psychologically burdening the consumer. At present, there is little research on packaging methods for fresh pecan kernels. Thus, it is necessary to explore simple packaging methods and evaluate the quality changes of fresh pecan kernels during storage first.

Overall, the aims of this study are: (1) comparing the mechanical shelling efficiency of fresh pecans with varying moisture contents to obtain suitable moisture content for future commercialization of fresh pecans; (2) evaluating the nutrients of fresh and dried pecans to obtain the nutritional features of fresh pecans; and (3) assessing the quality changes during the storage of fresh pecan kernels to obtain suitable packaging methods for fresh pecans.

## 2. Materials and Methods

### 2.1. Sample Collection and Preparation

Fresh pecan nuts (cultivar “Wichita”) were harvested from a commercial orchard in Xuzhou City, Jiangsu Province, China (33°43′~34°58′ N, 116°22′~118°40′ E) on 30 October, 2021, with a total weight of 300 kg. The harvesting standard was that the green husks had fully split, and the fruits were fully developed and mature. The fresh pecan nuts were transported to the laboratory as soon as possible after the green husk was removed. To assess their mechanical shelling efficiency, fresh nuts were dried in forced air driers (DHG-9140A, Shanghai Yiheng Scientific Instrument Co., Shanghai, China) at 32 °C for durations of 0 h, 2 h, 4 h, 8 h, 12 h, 24 h, and 48 h. To compare the nutritional features of fresh and dried pecans, three groups of conventionally treated dried pecans were used as controls: sun-dried (exposed to sunlight for 7 days, with daily sunlight exposure of 5–7 h and light intensity ranging from 30,000 to 60,000 Lux), shade-dried (spread out in the shade of the laboratory for 15 days), and roasted (roasted at 105 °C for 3 h).

Each group of fresh samples contained 4 kg of nuts and were shelled using a commercial sheller (Savage Equipment, Madill, OK, USA). Each kilogram of sample was used as one replicate, for four replicates. Testa color, moisture content, oil content, soluble sugar content, peroxide value (PV), acid value (AV), total phenolic content (TPC), and antioxidant capacity were examined after counting the shelling efficiency, along with sensory evaluation.

A standard nut and kernel evaluation system, used by the U.S. Department of Agriculture for over 70 years [22], was applied to assess mechanical shelling efficiency. The percentage of packing material retained in dorsal grooves (DGP%), the percentage of packing material retained in ventral grooves (VGP%), and the percentage of kernel shoulders damaged (DSh%) were recorded. Any remaining shells on the kernels were manually removed after mechanical shelling, and the time required to separate the shells per kilogram (Time) was also recorded. Kernel weight percentages were calculated as follows: the total kernel percentage (K%, kernel weight after manual removal/total weight before mechanical shelling) and the kernel percentage after mechanical shelling (ShK%, kernel weight after manual removal/total weight after mechanical shelling). The kernel pieces were then graded into four classes according to their sizes: complete halves (CH, >80% intact), large pieces (LP, 80% to 50% intact), small pieces (SP, 50% to 25% intact), and chips (C, <25% intact). Each class was weighed, and the percentages were calculated.

### 2.2. Packaging Methods

Four groups of pecan kernels with high mechanical shelling efficiency were selected for packaging and storage. Each group of pecan kernels was packaged using three different methods: polyethylene film packaging with air (PEA), aluminum film packaging with 98% nitrogen (ALN), and aluminum film packaging with vacuum (ALV). The aluminum film used in this study had a thickness of 100 μm, a water vapor transmission rate below 0.01 g·m^−2^·day^−1^, and an oxygen transmission rate below 0.1 cm^3^·m^−2^·day^−1^. The polyethylene film had a thickness of 80 μm, a water vapor transmission rate between 5 and 10 g·m^−2^·day^−1^, and an oxygen transmission rate between 1000 and 1500 cm^3^·m^−2^·day^−1^. Each package contained five complete halves and water absorbent as one replicate, stored at ambient temperature and atmospheric pressure. Three replicates were taken for each storage time. The testa color, moisture content, PV, AV, TPC, and antioxidant capacity were measured immediately and after 1, 3, 6, and 9 months of storage.

### 2.3. Appearance and Color Analysis

The appearance of the pecan testa was recorded under white light using a Canon EOS 5D camera (Canon, Tokyo, Japan). The color of the testa was measured with an NR10QC universal colorimeter (Shenzhen Sanenshi Intelligent Technology Co., Shenzhen, China), with the illuminant D65 and an angle of 8°. For unpackaged samples, four complete halves were randomly selected from each replicate, and for packaged samples, three complete halves were randomly selected from each package, and measurements were taken at four locations on the smoothest area of the dorsal side of each half. The color was recorded using the CIELab color model, which reflects brightness (L*), grayscale, and saturation (a*: red–green degree, b*: yellow–blue degree) [23]. The initial color data (0 h and day 0) served as the control. The total color difference (∆E), representing the perceptible color difference to the human eye, was calculated using the following Equation (1).

(1) $$\Delta E=\sqrt{{\left(\Delta L\right)}^{2}+{\left(\Delta a\right)}^{2}+{(\Delta b)}^{2}}$$

### 2.4. Moisture and Oil Content

The moisture content (wet basis) of the pecan kernels was determined according to GB 5009.3-2010 [24]. The kernels were ground in liquid nitrogen, and approximately 4 g of the ground sample were placed into a pre-dried weighing bottle. The samples were dried in an oven at 105 °C for 2–4 h, then cooled in a desiccator for 30 min before being weighed. This procedure was repeated until the weight difference between the two consecutive weightings did not exceed 0.002 g. The moisture lost was calculated based on the initial and final weights.

The oil content (wet basis) was determined using the Soxhlet extraction method as outlined in GB 5009.6-2016 [25]. The ground kernels were placed in filter paper cartridges tied with skim cotton thread and placed in a Soxhlet extractor, refluxed with an appropriate amount of petroleum ether for at least 6 h. The solvent was removed using a rotary evaporator, and the oil content was calculated by recording the weight of the sample and the receiving flask before and after extraction.

### 2.5. Fatty Acid Content

The fatty acid content of the pecan kernels was analyzed using gas chromatography after different drying treatments. Fatty acid methyl esters (FAMEs) were prepared according to GB 5009.168-2016 [26]. Approximately 300 mg of pecan oil were mixed with 8 mL of 2% sodium hydroxide methanol solution (NaOH/MeOH, *v*/*v*) and placed in a water bath at 80 °C under nitrogen protection. After the oil droplet disappeared, 7 mL of 15% trifluoro boron (methanol solution) were added, and the mixture was refluxed for 2 min. The flask was then cooled to ambient temperature and vibrated for 2 min after the heptane was added. Twenty milliliters of saturated sodium chloride solution were added to the flask, and the supernatant was collected and added to anhydrous sodium sulfate. The supernatant was collected for follow-up analysis after shaking vigorously and stewing.

FAMEs were analyzed according to previous reports [27] using an Agilent 6890N gas chromatograph (Supelco SP-2340 column, 100 m × 0.25 mm, 0.20 µm, Sigma-Aldrich, St. Louis, MO, USA). The initial temperature was 100 °C for 2 min, increased to 200 °C at a rate of 5 °C·min^−1^, maintained for 1 min, and then increased to 280 °C at a rate of 10 °C·min^−1^ and maintained for 10 min. The sample injection volume was 1.0 μL, and the injection and flame ionization detector temperatures were set to 250 °C and 200 °C, respectively. The flow rates of helium, air, and hydrogen were set at 1.6, 300, and 35 mL·min^−1^, respectively. Fatty acid content was determined by comparing retention times with the 37-component FAME mix standards and calculated using the area normalization method.

### 2.6. Soluble Sugar Content

Soluble sugar content was measured according to previous reports [28,29]. Briefly, 1 g of ground pecan kernel was dissolved in 80% ethanol and heated at 80 °C for 30 min. After centrifugation for 15 min at 10,000× *g*, the supernatant was collected, and the extraction was repeated twice. The combined supernatants were evaporated in a rotary evaporator and dissolved in 15 mL of boiling water. Then, 1 mL of the sample solution was mixed with 5 mL of anthrone reagent and boiled for 10 min. After cooling, absorbance was measured at 620 nm using a UV spectrophotometer (754PC, Shanghai Jinghua Technology Instrument Co., Shanghai, China). Different concentrations of glucose solutions were prepared to measure absorbance, and a standard curve equation was plotted as Equation (2).

(2) $$y=4.4822x-0.0056,\;R^{2\;}=0.9994$$

### 2.7. Peroxide Value and Acid Value

The PV and AV of pecan kernels were determined by titration according to GB 5009.227-2016 [30] and GB 5009.229-2016 [31]. To determine the PV, 2–3 g of pecan oil were dissolved in a mixture of methylene chloride and glacial acetic acid (2:3, *v*/*v*). The iodine produced by the reaction of peroxide in the oil with potassium iodide was titrated using a standard solution of sodium thiosulfate (0.002 mol·L^−1^). The volume of the standard solution consumed (V_1_) and the standard solution consumed for the blank experiment (V_0_) were recorded. The PV was calculated according to Equation (3).

(3) $$\mathrm{PV}\;\left(g\cdot {100g}^{-1}\right)=\frac{(V_{1}-V_{0})\times 0.002\times 0.1269}{\mathrm{weight}\;\mathrm{of}\;\mathrm{oil}}\times 100$$

For AV determination, 3 g of pecan oil were dissolved in a mixture of ethyl ether and isopropyl alcohol (1/1, *v*/*v*) with phenolphthalein indicator. The standard solution of potassium hydroxide (0.1 mol·L^−1^) was used to titrate. The titration was stopped when the reddish color appeared and did not fade significantly within 15 s. The consumption of standard titration solution volume was recorded as V_1_. The volume of the standard solution consumed for the blank experiment was recorded as V_0_. The AV of the sample was calculated according to Equation (4).

(4) $$\mathrm{AV}\;(\mathrm{mg}\cdot g^{-1}\;\mathrm{of}\;\mathrm{oil})=\frac{\left(V_{1}-V_{0}\right)\times 0.1\times 56.1}{\mathrm{weight}\;\mathrm{of}\;\mathrm{oil}}$$

### 2.8. Total Phenolic Content and Antioxidant Capacity

The TPC was measured according to previous reports with slight modifications [32]. Ground pecan kernels (0.2 g) were vortex-extracted with 5 mL of 80% ethanol. After resting for 2 h, the extracts were centrifuged for 10 min at 4000× *g*. One hundred microliters of supernatant were mixed with 2 mL of 7.5% Na_2_CO_3_ and 2.5 mL of 10% Folin phenol reagent. The absorbance was measured at 760 nm after 1 h of reaction in the dark. Eighty percent ethanol was used as the blank solution. Gallic acid was used as the standard material. Results were expressed as milligrams of gallic acid equivalent per gram of fresh kernel weight (mg·g^−1^); that is, the equivalent content of gallic acid contained in each gram of fresh kernel. According to the reaction results of the standard solution under different concentration gradients, the standard curve is drawn as Equation (5).

(5) $$y=2.0064x-0.0208,\;R^{2}=0.9959,$$

Antioxidant capacity was assessed using the 2,2-Diphenyl-1-picrylhydrazyl (DPPH) and 2,2′-Azino-bis (3-ethylbenzothiazoline-6-sulphonic acid) diammonium salt (ABTS) free-radical scavenging assays. DPPH scavenging ability was measured at 517 nm [33]. The DPPH radical solution (39.4 mg) was mixed with 80% ethanol to obtain the working solution (0.1 mmol·L^−1^). The sample ethanol extraction (10 μL) was mixed with 4 mL of DPPH working solution, and the absorbance was measured after reacting at room temperature in the dark for 30 min. The 80% ethanol solution without the sample was used as the blank control. Trolox was used as the standard material. The DPPH free-radical scavenging ability was expressed by mg Trolox equivalent antioxidant capacity per gram of fresh kernel weight (TE)·g^−1^. The standard curve was obtained with absorbance (y) and Trolox concentration (x) as Equation (6).

(6) $$y\;=-0.2836x+0.4752,\;R^{2}=0.9993,$$

ABTS scavenging ability was determined according to previous reports [34,35]. The ABTS (0.384 g) and potassium persulfate (0.134 g) were dissolved in distilled water (100 mL), respectively, and were mixed (1:1, *v*/*v*) and placed in the dark for 12 h to obtain the ABTS working solution. Before use, the solution should be diluted 40 times with 80% ethanol. The absorbance of the sample was measured at 760 nm immediately after the 2 μL ethanol extract was added to the 2 mL ABTS diluent. The 80% ethanol solution without the sample was used as a blank control. Trolox was used as the standard. The ABTS free-radical scavenging was expressed by μmol Trolox equivalent antioxidant capacity per gram of fresh kernel weight (TE)·g^−1^. The standard curve was obtained with absorbance (y) and Trolox concentration (x) as Equation (7).

(7) $$y=-0.5999x+0.9092,\;R^{2}=0.9966$$

### 2.9. Sensory Evaluation

Descriptive sensory analysis was used to evaluate the sensory characteristics of pecan kernels with different drying treatments. The sensory evaluation involved a panel consisting of five men and five women, aged between 22 and 42 years. The evaluation methodology was modified based on previous research [19,23,36]. Before the sensory evaluation, every assessor underwent at least 1 h of training to ensure familiarity with various attributes of the pecan kernel, including testa color, flesh color, moisture perception, fat perception, and overall flavor. A scale from 1 (lowest) to 9 (highest) was used to rate these traits. Kernels from different drying treatments were placed in separate plastic cups, with three complete halves for each treatment group. The groups were presented to the evaluators in random order. Panelists were asked to taste and rate the three kernels from each group within a 30 min period and leave at least a 30 min interval between tastings of different sample groups to minimize flavor residue. Each panelist evaluated 10 kernel samples in total. Each panelist’s rating of the three kernels from each group was considered one biological replicate. Sensory evaluation data were collected using paper forms, and the average score for each attribute was calculated.

### 2.10. Statistical Analysis

Data were recorded using Microsoft Excel 2016 (Microsoft©, Redmond, WA, USA). Graphs were generated using GraphPad Prism 9.0 (GraphPad Software Inc., La Jolla, CA, USA). Statistical analysis was performed using one-way analysis of variance (ANOVA) with IBM SPSS Statistics 26.0, and significant differences among treatments were identified. Tukey’s honest significant difference test was applied for post-hoc comparisons when ANOVA results showed significance (*p* < 0.05). Principal component analysis (PCA), radar plots, correlation analysis, and Spearman correlation coefficients were calculated using R 4.4.2.

## 3. Results

### 3.1. The Effect of Drying Time on the Mechanical Shelling Efficiency

The moisture content of pecan kernels significantly influences the mechanical shelling efficiency (Table 1). Both the DGP% and VGP% were relatively high at the beginning, followed by a gradual decline, reaching a minimum at 48 h. The DSh% also exhibited a general downward trend. The lowest level of DSh% was observed at 24 h of drying, followed by samples dried for 8 and 12 h, even lower than those of the 48 h dried samples. The K% showed a gradual increase, reaching its highest level when drying for 48 h. The kernel before drying exhibited the lowest ShK%, while the highest ShK% was observed after drying for 48 h. A lower value indicated a greater proportion of shells remaining after mechanical shelling, suggesting that the mechanical shelling process may have been less effective. The time required for manual separation was recorded to reflect the degree of mixing between shells and kernels. The overall trend for Time decreased as the drying time increased. Similar to Dsh%, the 24 h dried sample had the shortest manual separation time (10.67 min·kg^−1^), which was shoter than 48 h (16.33 min·kg^−1^). Meanwhile, the CH% gradually increased from 31.35 to 91.60 and then decreased to 75.74% at 48 h of drying. Among these, the CH% of pecan kernels dried for 24 h reached a maximum, and the LP%, SP%, and C% were also the lowest, indicating the highest integrity of the kernel.

As an important index of mechanical shelling efficiency, CH% is related to shell–kernel separation, and a high CH% can only be guaranteed when the shell-breaking deformation is less than the shell–kernel gap [37]. In commercial shellers with the same cracking strength, kernels with higher moisture content adhered tightly to the shells and were peeled during shelling, which led to a lower K%. After 48 h of drying, the packing material lost moisture, and the shell–kernel gap increased, but the kernels became too dry and brittle, resulting in an increase in DSh%. Moderate drying (drying for 24 h) reduced the moisture content in the packaging material and increased the shell–kernel gap, allowing the kernels to maintain a certain degree of elasticity. This facilitated the separation of the shell and kernel, resulting in the lowest DSh% and highest CH%. Similar to the study of McKay, the mechanical shelling efficiencies of nuts pre-treating with hot water or steam for 7 min before shelling were enhanced compared to those of the untreated group. The complete half rate of the kernel in that study averaged 43.3%, with a range of 29.5% to 52.8% [12]. In contrast, drying the pecan nuts for 24 h significantly improved shelling efficiency by 111.5% to 210.5%, surpassing the rate observed in the aforementioned study. This also suggests that retaining some moisture helps increase the efficiency of mechanical shelling, as increased moisture content makes the kernels more pliable and prevents them from breaking into pieces during cracking, shelling, and subsequent handling operations [38]. In summary, after 24 h of drying, a kernel moisture content of approximately 17.51% is considered the optimum for the commercial shelling of fresh pecans.

### 3.2. Appearance and Nutritional Features of Fresh Pecans

To evaluate the nutritional features of fresh pecans, fresh pecan kernels with different moisture contents were analyzed for their nutrient compositions and antioxidant capacities. The results were then compared with pecans dried by sun, shade, and roasting.

#### 3.2.1. Color Variations

**Figure 1:**
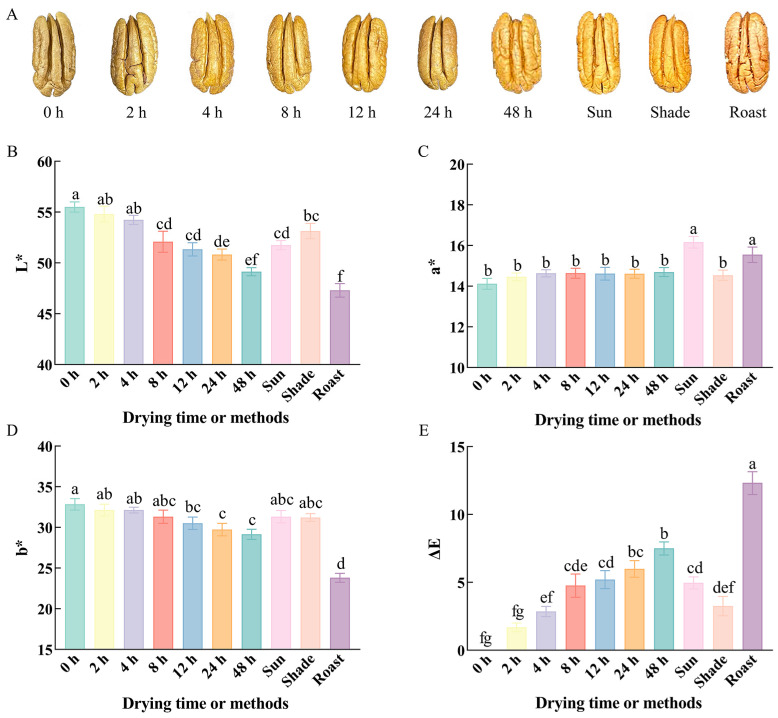
The testa color (**A**), L* (**B**), a* (**C**), b* (**D**), and ΔE (**E**) of pecan kernels with different drying time or methods. Different lowercase letters (a–g) indicate significant differences (*p* < 0.05) between samples.

**Figure 2:**
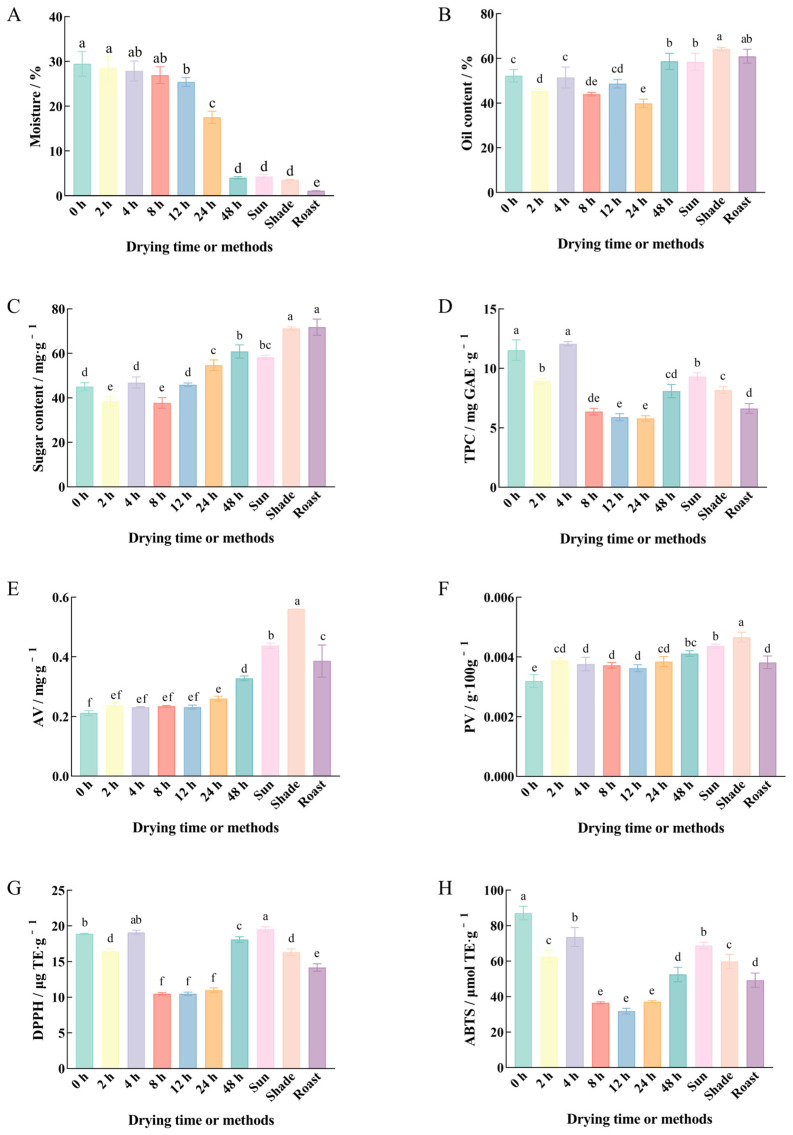
Influence of drying time or methods on the moisture content (**A**), oil content (**B**), sugar content (**C**), TPC (**D**), AV (**E**), PV (**F**), DPPH (**G**), and ABTS (**H**) of pecan kernels. Different lowercase letters (a–f) indicate significant differences (*p* < 0.05) between samples.

The color of the testa of fresh pecans changed after different drying times (Figure 1A). The brightness (L*) of fresh pecan kernels decreased rapidly during the first 12 h of drying, with little change observed during the subsequent 12–24 h, reaching a minimum at 48 h (Figure 2B). The changes in the parameters a* and b* exhibited more moderate trends. The parameter a*, which represents redness and greenness, did not vary significantly across samples with different drying times. In contrast, the overall trend of b*, which represents the yellow–blue degree, exhibited a gradual decline, which means the color was getting darker. The ΔE represents the perceptible color difference perceived by the human eye in a uniform color space. An increase in ΔE indicates that, with longer drying times, the color difference between the test sample and the before-drying sample becomes more pronounced. Among the methods used to obtain dried kernels, sun-drying significantly increased the parameter a*, while roasting significantly decreased both L* and b* and substantially increased ΔE, which means the greatest impact on the color of the testa. In contrast, shade-drying caused the least color change. The external browning caused by drying results from a combination of non-enzymatic reactions, surface dehydration, and caramelization, with phenolic compounds in the testa often influencing the extent of browning [39,40]. During development, certain phenolic compounds accumulate in the testa, acting as brown pigment precursors and promoting browning, particularly in nuts harvested at later stages of development, which also increases the susceptibility of the kernels to browning during storage [41]. Early research suggested that consumers are more likely to purchase pecans with lighter colors and are even willing to pay a higher price range for early harvested kernels [42]. The selection of fruits at the appropriate developmental stage is crucial for the commercialization of fresh pecans.

#### 3.2.2. Nutrient Contents and Antioxidant Capacities

Before drying, fresh pecan kernels may contain up to 29.47% moisture (Figure 2A). At the beginning of drying, the moisture content decreased gradually, reaching 25.45% after 12 h of drying, 17.51% after 24 h of drying, and 4.02% after 48 h of drying, which was the same as the moisture content of the sun-dried kernels. At this point, the moisture content met the required commercial standards.

The oil content of fresh kernels is generally lower than that of dried kernels (Figure 2B). After 48 h of oven drying, the oil content of fresh pecans was close to that of dried pecans with different drying methods. Oil content affects the texture when people eat it, with higher oil content resulting in a fattier mouthfeel. Similarly, the soluble sugar content was affected by the reduction in moisture content (Figure 2C). The soluble sugar content of fresh pecans showed an overall increase with the duration of drying. After drying, the TPC of fresh pecan kernels gradually decreased (Figure 2D).

The AV and PV of fresh pecan kernels were significantly lower than those of dried pecan kernels (Figure 2E,F), indicating higher quality for consumption. Among the drying methods, the PV of pecan kernels obtained by shade drying was the highest, which may be attributed to the increased oxidative reactions resulting from prolonged exposure to shade drying. Pecan kernels are rich in phenolic compounds, which possess antioxidant capacity. In parallel with TPC, the antioxidant capacity, as measured by DPPH and ABTS free-radical scavenging assays, showed a similar decreasing trend. Before drying, the TPC and antioxidant capacity of fresh pecan kernels were significantly higher than those of dried pecan kernels. These findings are consistent with those of Rábago–Panduro et al., who reported that the drying process reduced the phenolic content and antioxidant capacity of pecan kernels [43]. Similarly, the TPC in fresh walnuts has been found to be 1.2 times higher than that in dried walnuts [44]. The loss of phenolic compounds during processing, especially thermal processing, is unavoidable.

Pecan is one of the famous woody oil crops, known for its high oil content. The composition of fatty acids in its kernels has an important effect on kernel quality. A total of 14 fatty acids, with concentrations greater than 0.01%, have been identified in pecan kernels, including six saturated fatty acids (SFAs) and eight unsaturated fatty acids (UFAs) (Table 2 and Figure S1). The fatty acid composition varied slightly across different drying methods. Oleic acid (50.62–58.27%) was the most abundant fatty acid, followed by linoleic acid (30.17–37.97%). The monounsaturated fatty acid (MUFA) content of kernels before drying, particularly oleic acid (58.27%), was significantly higher than that of other groups, which aligns with Rábago–Panduro et al. Furthermore, oleic acid content was found to be 1.6 times higher than previous results [43]. In contrast, the SFA content of fresh pecan kernels was significantly lower than that of shade-dried pecan kernels. A diet rich in MUFAs can reduce serum total cholesterol, low-density lipoprotein cholesterol, and triglyceride levels while increasing high-density lipoprotein cholesterol, thus improving the lipid profile and reducing cardiovascular risk [45]. Replacing SFA calories with MUFAs, rather than carbohydrates, may have beneficial effects on reducing cardiovascular disease risk [46]. This result also suggests that fresh pecans have a higher nutritional value potential than dried pecans. In addition to its health benefits, subtle differences in fatty acid profiles may also impact the flavor of the nut kernel. For example, palmitic acids and stearic acids are negatively correlated with sourness, while linoleic acid is positively correlated with sourness [36].

#### 3.2.3. Sensory Evaluation Analysis

**Figure 3:**
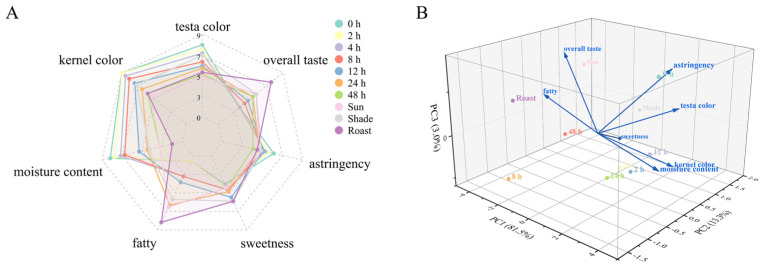
The radar plot (**A**) and principal component analysis (PCA), (**B**) of sensory descriptor scores of pecan kernels with different drying times or methods. PC (principal components).

The sensory evaluation results demonstrated that the color of the testa of fresh pecan kernels before drying received the highest score (Figure 3A). As the drying time progressed, the color of the testa gradually transitioned from bright yellow to brownish yellow, accompanied by a corresponding decline in the sensory evaluation scores. The color of the testa of shade-dried pecan kernels showed less variation and received higher sensory scores than fresh pecan kernels dried for 8 h. However, the color of the kernel itself did not show a clear advantage, which was lower than those of the 12 h drying samples but higher than those of the 24 h drying samples. This may be due to the long drying time causing signs of oil leakage and opalescence inside the kernels [47]. Overall, the sensory evaluation scores for the color of the kernels inside the testa declined gradually as the drying time increased. Similar to the results of moisture determination experiments, undried pecan kernels scored the highest for moisture sensation and the lowest for fat sensation, suggesting that fresh pecan kernels are expected to provide a fresher and crisper eating experience. Roasted pecan kernels exhibited the lowest moisture perception score and the highest edible fat perception score, which may contribute to an increased sense of greasiness during consumption. The highest sweetness scores were observed in the shade-dried and roasted samples. The highest levels of bitterness and astringency were found in fresh pecan kernels dried for 48 h. The PCA output revealed that the undried kernels were distinctly characterized by high moisture and low astringency (Figure 3B). Roasted pecan kernels exhibited a distinctive flavor profile, influenced by the high-temperature roasting process. This is due to the Maillard reaction that occurs during the roasting, which results in the formation of pyrazines and an increase in lipid-derived hydrocarbons. These compounds significantly alter the volatile characteristics of the pecan, adding a unique organoleptic profile, while different pecan varieties exhibit distinct flavor profiles [48,49]. However, the roasting process also masked the inherent sweetness of the pecan kernels.

The first two principal components (PC1 and PC2) explained 94.8% of the total variance, effectively condensing the sensory data into a lower-dimensional space. A cumulative principal component greater than 85% is sufficient to account for the dataset’s overall variance [50]. According to an analysis of the loadings for PC1 and PC2, PC1 is mainly driven by moisture content and fat, in that it increases with increasing moisture content and decreasing fat. Moreover, overall flavor was linked to negative loadings on PC1, but testa, kernel color, and astringency were substantially associated with positive loadings on it. PC2 distinguished between the samples according to their astringency, moisture content, and testa and kernel color.

The overall taste evaluation, combining all indicator factors and the personal preferences of the assessment team, was conducted. The ratings showed a bifurcation. Some panelists preferred roasted pecans’ flavor, as roasting enhanced the aroma of the oils, while unroasted pecans have a bland taste. On the other hand, some panelists reported that fresh kernels were perceived as more refreshing, sweet, and crunchy, with a creamy flavor, considering roasted pecans to be greasy. It provides them a new perspective on pecans. This means that fresh pecans have a certain potential audience in the market.

### 3.3. Effect of Different Packaging Methods on Storage

Four groups of samples, dried for 8 h, 12 h, 24 h, and 48 h, respectively, were selected for packaging and storage experiments due to their relatively high mechanical shelling efficiencies, similar nutritional profiles, and comparable taste, with all samples having a CH% above 50%.

#### 3.3.1. Changes of Color During Storage

**Figure 4:**
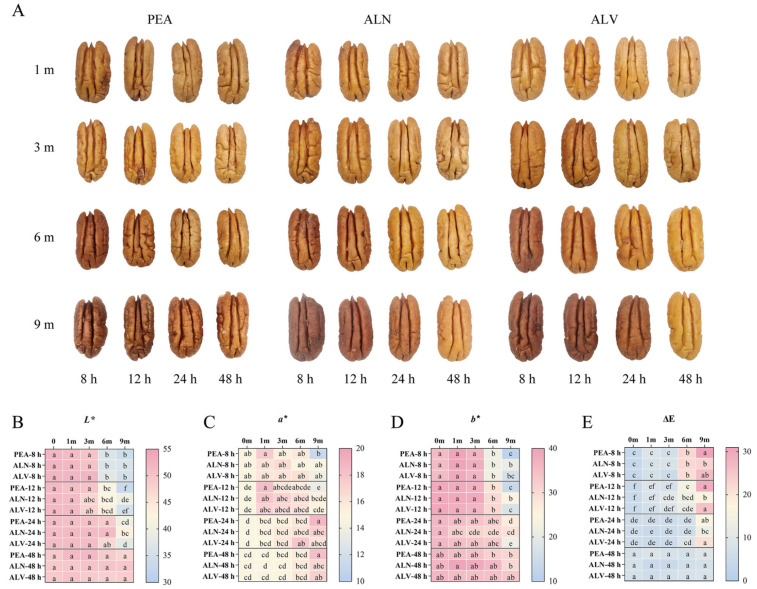
Changes in the testa color (**A**), L* (**B**), a* (**C**), b* (**D**), and ΔE (**E**) of pecan kernels with four different drying times and three different package methods during 9 months of storage. Different lowercase letters (a–f) indicate significant differences (*p* < 0.05) between samples of the same drying time.

The testa color shifted from light yellow to reddish brown during prolonged storage. (Figure 4A). Following long-term storage, the L* and b* parameters decreased, and lower values indicated deeper browning (Figure 4B,D). However, the a* parameter of the samples dried for 8 and 12 h initially increased and then decreased, while it gradually increased in the samples dried for 24 and 48 h (Figure 4C). Additionally, the ΔE value increased over time (Figure 4E). Among these, the L* and b* were the primary parameters affected by storage time, whereas the a* parameter, which symbolizes redness, may also be affected by other factors such as anthocyanins [51]. After 6 months of storage, the samples dried for 8 and 12 h showed a significant change in testa color, especially those packaged in PEA. The samples dried for 24 h showed considerable browning after 9 months of storage. As expected, the samples dried for 48 h showed the least color change during storage. Consistent with the results of this study, in-shell macadamia nuts with a moisture content of 25% exhibited a higher degree of browning in the center of the kernel compared to samples with lower moisture content during storage [52]. Previous studies have indicated that testa color is influenced by storage temperature, humidity, oxygen partial pressure, and variety [53,54,55]. The results of this study revealed that the storage stability of pecan testa was also affected by their moisture content: the higher the initial moisture content of the sample, the faster the testa color change.

Among the samples with the same moisture content, those packaged in PEA showed the most browning, while those packaged in ALN showed the least color change. At a higher moisture content of the kernel, ALN most effectively delayed the browning of kernel testa. Yang et al. confirmed that kernel appearance impacts consumer acceptance [56], and that regulating relative humidity and oxygen partial pressure through packaging and storing at low temperatures can delay the darkening of the testa color [57,58]. Traditionally, testa color has been used as a measure of the overall quality of the kernel, and lighter-colored testa are preferred over darker ones in sales. Therefore, when studying fresh pecans, more emphasis should be placed on color changes in the testa.

#### 3.3.2. Changes of Nutrients During Storage

**Figure 5:**
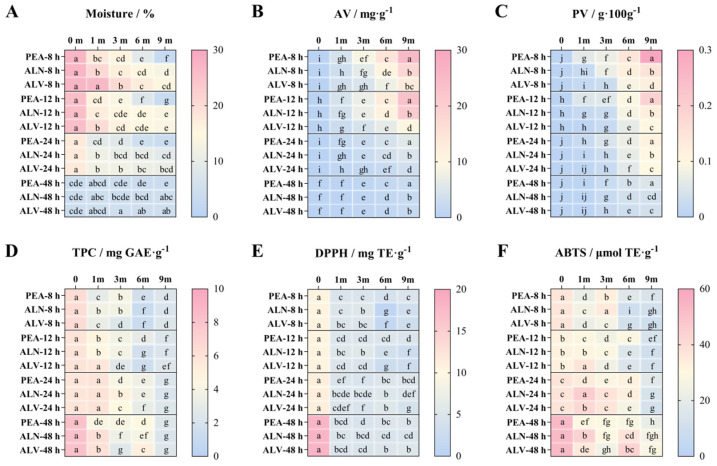
Changes in the moisture (**A**), AV (**B**), PV (**C**), TPC (**D**), DPPH (**E**), and ABTS (**F**) of pecan kernels with four different drying times and three different package methods during 9 months of storage. Different lowercase letters (a–j) indicate significant differences (*p* < 0.05) between samples of the same drying time.

The moisture content of fresh pecan kernels gradually decreased during storage (Figure 5A). Samples dried for 8 h showed the most significant reduction in moisture content, decreasing from an initial 26.92% to 3.02% by the 9th month for samples packaged in PEA and 15.46% for those packed in ALV. A similar trend was observed in samples dried for 12 and 24 h. In contrast, the samples dried for 48 h showed minimal moisture change during storage, maintaining values between 3.65% and 4.83%. Even with the addition of an absorbent, ALV packaging demonstrated the best water retention properties among the three packaging methods.

The AV of all samples increased during storage (Figure 5B). The AV of samples dried for 8 h, 12 h, and 24 h increased significantly in the 1st month of storage. The AV of samples dried for 8 and 12 h increased more quickly than that of the samples dried for 24 h, exceeding the national standard of 3 mg·g^−1^ according to GB 2716-2018 [59] after the 1st month of storage. In contrast, the AV of the samples dried for 24 h and packed in PEA and ALN exceeded 3 mg·g^−1^ by the 6th month of storage, whereas the AV of those packed in ALV surpassed this threshold in the 9th month of storage, demonstrating that ALV packaging effectively extends the shelf life of samples dried for 24 h from 3 to 6 months. Samples dried for 48 h showed the slowest increase in AV, with a significant increase in the 3rd month and reaching values above 2 mg·g^−1^ by the 9th month. ALV packaging assisted in slowing the increase in AV across all four sample groups. The increase in AV is attributed to the breakdown of triglycerides into free fatty acids by lipases, and this hydrolysis process is more pronounced in kernels with higher moisture content than in those affected by high-temperature accelerated oxidation [60]. In addition to environmental factors and kernel moisture content, treatments with antioxidants such as 1-methylcyclopropene and sodium erythorbate can also help delay lipid hydrolysis [61,62].

The trend in PV followed a pattern similar to AV (Figure 5C). For the samples dried for 8 h, those packaged in PEA and ALN exceeded 0.1 g × 100 g^−1^ by the 6th month of storage, while those dried for 12 and 24 h exceeded this threshold by the 9th month. ALV packaging has proven to be relatively effective in preserving fresh kernels and could somewhat delay the deterioration of fresh kernels in the short term. This is likely because ALV packaging prevents the effects of both light and N_2_ on the seed kernels, compared to PEA and ALN. Mexis et al. demonstrated that raw almonds packed under N_2_ and light conditions exhibited higher PV, whereas in the presence of an oxygen absorber, PV was unaffected by light conditions [21]. Higher storage temperatures contribute to an increase in acidity, while lower oxygen partial pressures help to slow the oxidation process [58].

The TPC of all samples dried for 8 h decreased significantly in the 1st month of storage, and the same pattern was observed in the 12 h samples packed in PEA and ALN, as well as in all samples dried for 48 h (Figure 5D). However, no significant change was observed in the 24 h dried samples, although a significant decline began from the 3rd month of storage. A significant decrease in DPPH radical scavenging capacity was observed in all samples during the 1st month, and the levels stabilized between 1 and 5 mg TE·g-1 in the later stages of storage (Figure 5E). The trends of ABTS radical scavenging capacity during storage were similar to the TPC (Figure 5F), with the 24 h dried samples showing the slowest decline. Of the three packaging methods, ALN provided some protection against the loss of antioxidant capacity. The decline in TPC during storage may be attributed to a chain mechanism in which tocopherols act as hydrogen donors during autoxidation to prevent autoxidation [63]. Based on these findings, walnut green hulls, which contain various phenolics, can enhance the positive effects of chlorine dioxide in preserving the antioxidant capacity of kernels during storage [64].

#### 3.3.3. Correlation Analysis

**Figure 6:**
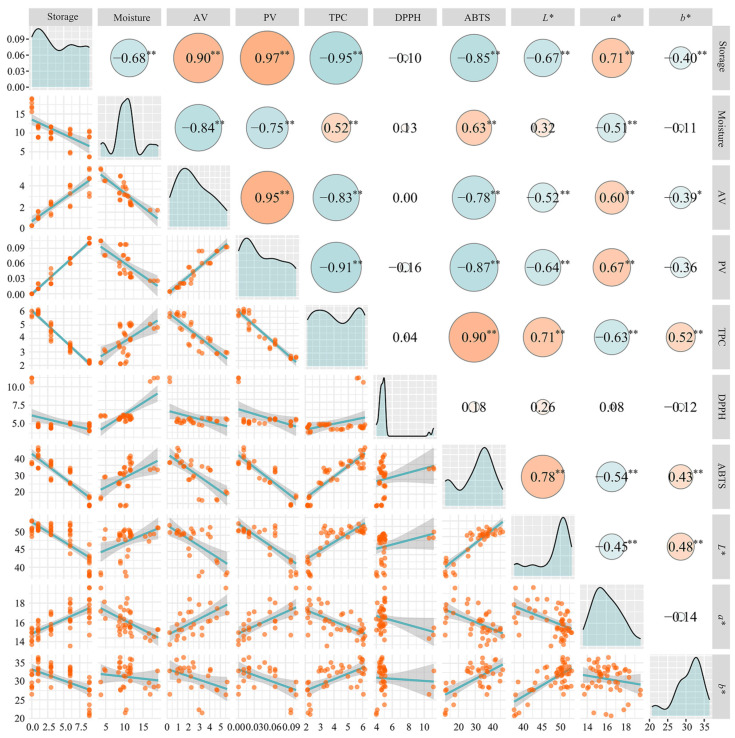
Correlation of quality characteristics of pecans dried for 24 h during storage. The size of the circles represents the absolute value of the correlation coefficient, with red indicating a positive correlation and blue indicating a negative correlation. * indicates significant differences (*p* < 0.05) between samples. ** indicates significant differences (*p* < 0.01) between samples.

The initial moisture content significantly affected the correlations between the degree of hydrolytic oxidation, antioxidant capacity, and testa color of pecan kernels during storage. There was a consistently significant positive correlation between storage time and the degree of hydrolytic oxidation (R > 0.90, *p* < 0.01), with the highest negative correlation between storage time and TPC observed in the 24 h dried samples (R = 0.95, *p* < 0.01) (Figure 6). Storage time showed significant negative correlations with the parameters L* and b* of samples dried for 8 and 12 h (Figures S2 and S3), while it exhibited significant positive correlations with the parameter a* for samples dried for 24 and 48 h (Figure S4). This indicates that the effect of storage time on the parameters L* and b* is more pronounced when the initial moisture content is higher, while the effect on a* is greater when the initial moisture content is lower. Thus, changes in testa color can be used to estimate the storage time of pecan kernels and to compare the freshness of the kernels.

A significant negative correlation (R > 0.91, *p* < 0.01) was observed between moisture content during storage and the degree of oxidation in samples dried for 8 and 12 h, alongside a positive correlation with the antioxidant capacity of the kernels. A similar positive correlation was observed between the moisture content of fresh walnuts (24.23% moisture content) stored at 25 °C for 12 days and their TPC and DPPH radical scavenging capacity [64]. As the initial moisture content of the pecan kernels decreased during storage, the correlations between moisture content, storage time, degree of hydrolytic oxidation, antioxidant capacity, and testa color gradually weakened. In samples dried for 48 h, these correlations were nearly nonexistent. This suggests that the degree of hydrolytic oxidation is significantly affected by moisture content in pecan kernels with higher initial moisture, whereas in kernels with low moisture content, the change in moisture content during storage is minimal, thus having little effect on other traits.

The significant correlation observed in the samples with higher moisture content may be attributed to water, which serves as a medium for various life activities. Under high temperature and moisture content conditions, metabolism is accelerated, leading to faster exchange and oxidation reactions. In contrast, low moisture content slows metabolism and prolongs the storage time of pecan kernels.

## 4. Conclusions

This study systematically explored the commercial potential of fresh pecans for the first time by evaluating moisture content for the highest mechanical shelling efficiency, screening nutritional features by comparing fresh to dried pecans, and identifying suitable packaging methods. Fresh pecans with a moisture content of approximately 17.51% after 24 h of drying exhibited the highest CH% of 91.60%, the lowest DSh% of 31.70%, and the shortest manual separation time of 10.67 min·kg^−1^, which is the most suitable for mechanical shelling. Compared to dried pecans, freshly harvested pecans displayed brighter testa color, higher TPC, and superior antioxidant capacity, with a high oleic acid content of 58.27%, the highest percentage of MUFA, and lower oil content. The elevated moisture content of fresh pecans contributes to a more refreshing flavor and reduces the perception of greasiness, providing a healthier option for consumers. Furthermore, pecans dried for 24 h maintained quality for up to 3 months under PAE/ALN packaging and 6 months under ALV packaging. Fresh pecans dried for 24 h are suitable for commercial development, and distributors can choose ALV as a packaging method for fresh pecans. The results of the study emphasize the significant impact of moisture content on kernel quality, highlighting the importance of selecting fresh pecan kernels with suitable moisture content. This study provides a reliable foundation for the future commercialization of fresh pecan kernels. Due to the large range of drying time in this study, future research could further investigate the optimal moisture content for shelling fresh pecans within a drying time range of 12 to 48 h. The effects of temperature and various packaging methods on the storage of fresh pecans warrant further exploration.

## Figures and Tables

**Table 1:** The moisture contents and mechanical shelling efficiencies of pecan nuts with different drying times.

Drying Time	0 h	2 h	4 h	8 h	12 h	24 h	48 h
Moisture (%)	29.47 ± 2.76 a	28.5 ± 2.65 ab	27.85 ± 2.23 ab	26.92 ± 1.86 bc	25.4 ± 0.99 c	17.51 ± 1.37 d	4.02 ± 0.27 e
DGP (%)	62.00 ± 2.48 b	81.50 ± 3.67 a	34.40 ± 1.78 d	57.50 ± 1.71 c	36.50 ± 1.93 d	21.70 ± 2.23 e	14.50 ± 0.98 f
VGP (%)	62.01 ± 1.21 c	80.20 ± 2.90 a	68.80 ± 2.68 b	57.50 ± 2.84 d	36.50 ± 2.09 e	18.30 ± 1.25 f	13.20 ± 1.71 g
DSh (%)	76.00 ± 2.27 a	69.10 ± 3.07 b	65.60 ± 3.18 b	57.50 ± 1.46 c	47.60 ± 2.55 d	31.70 ± 1.32 e	65.80 ± 1.76 b
K (%)	40.72 ± 3.47 d	43.15 ± 2.22 d	57.69 ± 2.76 bc	54.24 ± 3.03 c	60.02 ± 2.18 b	56.47 ± 4.09 bc	70.89 ± 2.85 a
ShK (%)	54.92 ± 1.93 b	57.50 ± 4.31 ab	57.39 ± 2.70 ab	55.89 ± 3.31 ab	59.12 ± 4.26 ab	59.67 ± 2.08 ab	62.26 ± 4.46 a
Time (min·kg^−1^)	38.67 ± 2.52 b	52.67 ± 4.04 a	38.00 ± 2.00 b	28.67 ± 2.52 c	28.33 ± 1.53 c	10.67 ± 0.58 e	16.33 ± 1.53 d
CH (%)	31.35 ± 2.08 f	47.36 ± 1.70 e	44.58 ± 3.55 e	53.24 ± 2.29 d	63.72 ± 0.94 c	91.60 ± 4.61 a	75.74 ± 2.19 b
LP (%)	27.87 ± 1.89 a	21.18 ± 1.83 b	23.52 ± 1.04 b	15.38 ± 1.19 c	21.15 ± 1.80 b	4.83 ± 0.35 e	11.06 ± 0.45 d
SP (%)	16.15 ± 0.62 a	16.57 ± 1.14 a	16.32 ± 0.96 a	10.07 ± 0.82 b	6.37 ± 0.53 c	2.56 ± 0.18 d	5.92 ± 0.35 c
C (%)	24.64 ± 1.67 a	14.89 ± 0.54 c	15.58 ± 0.44 c	21.31 ± 0.87 b	8.75 ± 0.53 d	0.99 ± 0.03 f	7.29 ± 0.58 e ^1^

^1^ DGP (the percentage of packing material retained in dorsal grooves), VGP (the percentage of packing material retained in ventral grooves), DSh (kernel shoulders damaged), K (the total kernel percentage), ShK (the kernel percentage after mechanical shelling), Time (the times required to separate the shells per kilogram), CH (complete halves), LP (large pieces), SP (small pieces), and C (chips). Values are the mean ± SD. Means with different letters in the same row are significantly different (*p* < 0.05).

**Table 2:** The fatty acid compositions of pecan kernels with different drying times or methods (%).

	0 h	2 h	4 h	8 h	12 h	24 h	48 h	Sun	Shade	Roast
C14:0	0.18 ± 0.01 b	0.09 ± 0.00 f	0.08 ± 0.00 g	0.07 ± 0.00 g	0.14 ± 0.01 c	0.02 ± 0.00 h	0.12 ± 0.00 d	0.12 ± 0.00 e	0.21 ± 0.01 a	0.12 ± 0.00 e
C16:0	6.83 ± 0.12 b	6.63 ± 0.17 bc	6.42 ± 0.11 c	6.78 ± 0.17 b	6.80 ± 0.12 b	6.82 ± 0.21 b	6.83 ± 0.11 b	6.66 ± 0.17 bc	7.30 ± 0.16 a	6.76 ± 0.11 b
C16:1	0.11 ± 0.00 a	0.09 ± 0.01 cd	0.08 ± 0.00 cde	0.09 ± 0.00 c	0.11 ± 0.01 a	0.10 ± 0.00 b	0.08 ± 0.00 de	0.09 ± 0.00 cd	0.10 ± 0.01 b	0.08 ± 0.00 e
C17:0	0.06 ± 0.00 b	0.02 ± 0.00 f	0.04 ± 0.00 c	0.03 ± 0.00 e	0.07 ± 0.00 a	0.06 ± 0.00 b	0.04 ± 0.00 c	0.06 ± 0.00 b	0.04 ± 0.00 c	0.04 ± 0.00 d
C17:1	0.03 ± 0.00 ef	0.03 ± 0.00 fg	0.03 ± 0.00 g	0.06 ± 0.00 a	0.04 ± 0.00 c	0.04 ± 0.00d	0.03 ± 0.00 def	0.05 ± 0.00 b	0.03 ± 0.00 de	0.03 ± 0.00 de
C18:0	2.14 ± 0.07 bc	1.99 ± 0.05 de	2.02 ± 0.02 cd	1.86 ± 0.07 e	2.25 ± 0.06 ab	1.97 ± 0.04 de	2.30 ± 0.13 a	2.03 ± 0.03 cd	2.22 ± 0.10 ab	2.07 ± 0.09 cd
C18:1	58.27 ± 0.51 a	50.62 ± 1.23 c	56.55 ± 1.12 ab	50.86 ± 1.26 c	54.41 ± 2.12 b	54.12 ± 1.77 b	56.09 ± 0.34 ab	55.66 ± 1.11 b	50.95 ± 0.69 c	51.14 ± 1.65 c
C18:2	30.17 ± 0.29 d	37.97 ± 1.45 a	32.61 ± 0.80 c	37.01 ± 0.87 a	33.58 ± 0.77 bc	34.42 ± 1.00 b	32.08 ± 0.33 c	32.96 ± 1.09 bc	36.51 ± 0.75 a	37.28 ± 0.93 a
C20:0	0.11 ± 0.00 b	0.11 ± 0.00 b	0.11 ± 0.00 b	0.10 ± 0.00 c	0.11 ± 0.00 b	0.11 ± 0.00 b	0.12 ± 0.00 a	0.11 ± 0.00 b	0.11 ± 0.00 b	0.11 ± 0.00 b
C20:1	0.46 ± 0.01 c	0.48 ± 0.01 c	0.51 ± 0.01 bc	2.15 ± 0.10 a	0.46 ± 0.02 c	0.46 ± 0.01 c	0.51 ± 0.01 bc	0.56 ± 0.01 b	0.49 ± 0.02 c	0.46 ± 0.02 c
C21:0	0.02 ± 0.00 c	0.02 ± 0.00 d	0.02 ± 0.00 d	0.02 ± 0.00 d	0.02 ± 0.00 a	0.02 ± 0.00 b	0.02 ± 0.00 e	0.02 ± 0.00 e	0.02 ± 0.00 f	0.02 ± 0.00 c
C20:2	0.03 ± 0.00 d	0.03 ± 0.00 bc	0.03 ± 0.00 cd	0.03 ± 0.00 d	0.03 ± 0.00 d	0.03 ± 0.00 ab	0.03 ± 0.00 bc	0.03 ± 0.00 bc	0.03 ± 0.00 cd	0.03 ± 0.00 a
C20:3	0.10 ± 0.00 d	0.13 ± 0.00 b	0.10 ± 0.00 de	0.14 ± 0.01 a	0.10 ± 0.00 e	0.08 ± 0.00 f	0.08 ± 0.00 f	0.08 ± 0.00 f	0.11 ± 0.00 c	0.10 ± 0.00 de
C20:4	0.03 ± 0.00 b	0.03 ± 0.00 d	0.02 ± 0.00 f	0.02 ± 0.00 e	0.03 ± 0.00 c	0.04 ± 0.00 a	0.02 ± 0.00 d	0.02 ± 0.00 d	0.03 ± 0.00 d	0.02 ± 0.00 f
MUFA	58.88 ± 0.51 a	51.22 ± 1.25 e	57.17 ± 1.12 ab	53.15 ± 1.33 de	55.03 ± 2.12 bcd	54.72 ± 1.77 cd	56.71 ± 0.35 abc	56.36 ± 1.12 bc	51.57 ± 0.68 e	51.71 ± 1.63 e
PUFA	30.33 ± 0.29 d	38.16 ± 1.45 a	32.76 ± 0.80 c	37.20 ± 0.87 a	33.73 ± 0.77 bc	34.57 ± 1.00 b	32.22 ± 0.33 c	33.10 ± 1.09 bc	36.68 ± 0.75 a	37.43 ± 0.93 a
UFA	89.21 ± 0.58 a	89.38 ± 1.06 a	89.93 ± 0.92 a	90.35 ± 1.90 a	88.76 ± 2.76 a	89.29 ± 1.98 a	88.93 ± 0.34 a	89.46 ± 0.54 a	88.25 ± 1.39 a	89.13 ± 2.53 a
SFA	9.34 ± 0.19 bc	8.85 ± 0.14 de	8.68 ± 0.12 e	8.86 ± 0.20 de	9.39 ± 0.09 b	8.99 ± 0.25 cde	9.44 ± 0.24 b	9.00 ± 0.16 cde	9.89 ± 0.27 a	9.11 ± 0.18 bcd ^1^

^1^ MUFA (monounsaturated fatty acid), PUFA (polyunsaturated fatty acid), UFA (unsaturated fatty acid), SFA (saturated fatty acid). Values are the mean ± SD. Means with different letters in the same row for the same parameter are significantly different (*p* < 0.05).

## Data Availability

The original contributions presented in the study are included in the article/Supplementary Material. Further inquiries can be directed to the corresponding author.

## Supplementary Materials

The following supporting information can be downloaded at: https://www.mdpi.com/article/10.3390/foods14050757/s1, Figure S1: The GC chromatogram of pecan kernels; Figure S2: Correlation of quality characteristics of pecan dried for 8 h during storage. * indicates significant differences (*p* < 0.05) between samples. ** indicates significant differences (*p* < 0.01) between samples; Figure S3: Correlation of quality characteristics of pecan dried for 12 h during storage. * indicates significant differences (*p* < 0.05) between samples. ** indicates significant differences (*p* < 0.01) between samples; Figure S4: Correlation of quality characteristics of pecan dried for 48 h during storage. * indicates significant differences (*p* < 0.05) between samples. ** indicates significant differences (*p* < 0.01) between samples.

## Abbreviations

The following abbreviations are used in this manuscript:

DSh	Kernel shoulders damaged
CH	Complete halves
DPPH	2,2-Diphenyl-1-picrylhydrazyl
ABTS	2,2′-Azino-bis (3-ethylbenzothiazoline-6-sulphonic acid) diammonium salt
AV	Acid value
PV	Peroxide value
ALV	Aluminum film packaging with vacuum
TPC	Total phenolic content
DGP	The percent of packing material retained in dorsal grooves
VGP	The percent of packing material retained in ventral grooves
Time	The time required to separate the shells per kilogram
K	The total kernel percent
ShK	The kernel percent after manually removed
LP	Large pieces
SP	Small pieces
C	Chips
PEA	Polyethylene film packaging with air
ALN	Aluminum film packaging with nitrogen
FAMEs	Fatty acid methyl esters
PCA	Principal component analysis
ANOVA	One-way analysis of variance
SFA	Saturated fatty acid
UFA	Unsaturated fatty acid
MUFA	Monounsaturated fatty acid
PUFA	Polyunsaturated fatty acids
