# Supplementary material for: Effect of Moisture Content of Fresh Pecans on Mechanical Shelling Efficiency, Nutritional Features, and Packaging Method
Source: Foods. 2025 Feb 23;14(5):757. doi: 10.3390/foods14050757 (PMC11899440; doi:10.3390/foods14050757)
Supplement: Supplementary file 1 [file foods-14-00757-s001.zip › Supplementary Materials.pdf]

Supplementary Materials: The following supporting information can be downloaded at: www.mdpi.com/xxx/s1, Figure S1: The GC chromatogram of pecan kernels; Figure S2: Correlation of quality characteristics of pecan dried for 8 h during storage. * indicate significant differences ($p < 0.05$) between samples. ** indicate significant differences ($p < 0.01$) between samples; Figure S3: Correlation of quality characteristics of pecan dried for 12 h during storage. * indicate significant differences ($p < 0.05$) between samples. ** indicate significant differences ($p < 0.01$) between samples; Figure S4: Correlation of quality characteristics of pecan dried for 48 h during storage. * indicate significant differences ($p < 0.05$) between samples. ** indicate significant differences ($p < 0.01$) between samples.
