# Supplementary figures and images for: Effect of Moisture Content of Fresh Pecans on Mechanical Shelling Efficiency, Nutritional Features, and Packaging Method
Source: Foods. 2025 Feb 23;14(5):757. doi: 10.3390/foods14050757 (PMC11899440; doi:10.3390/foods14050757)

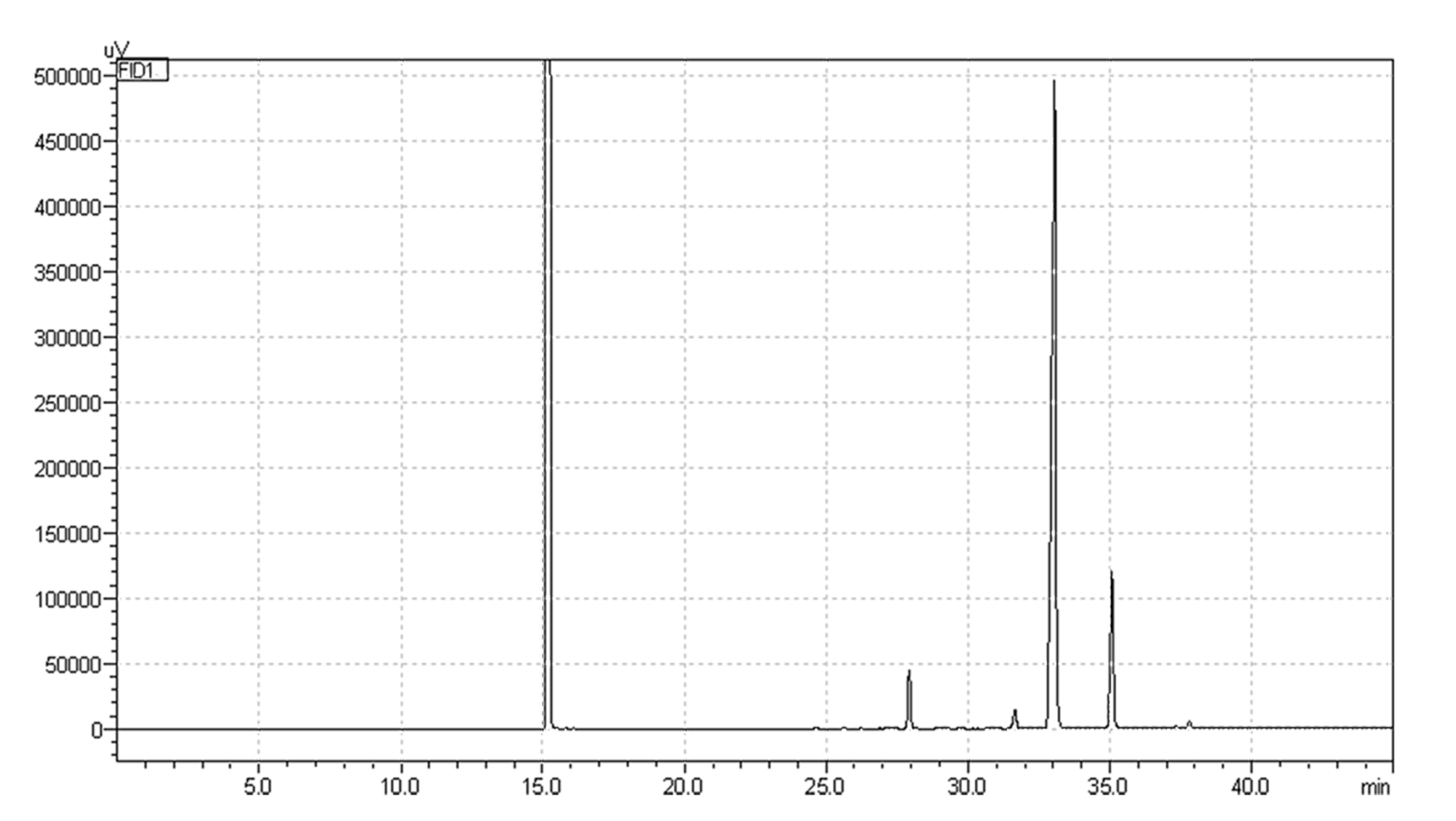

Supplement: Supplementary file 1 [file foods-14-00757-s001.zip › Figure S1.tif]

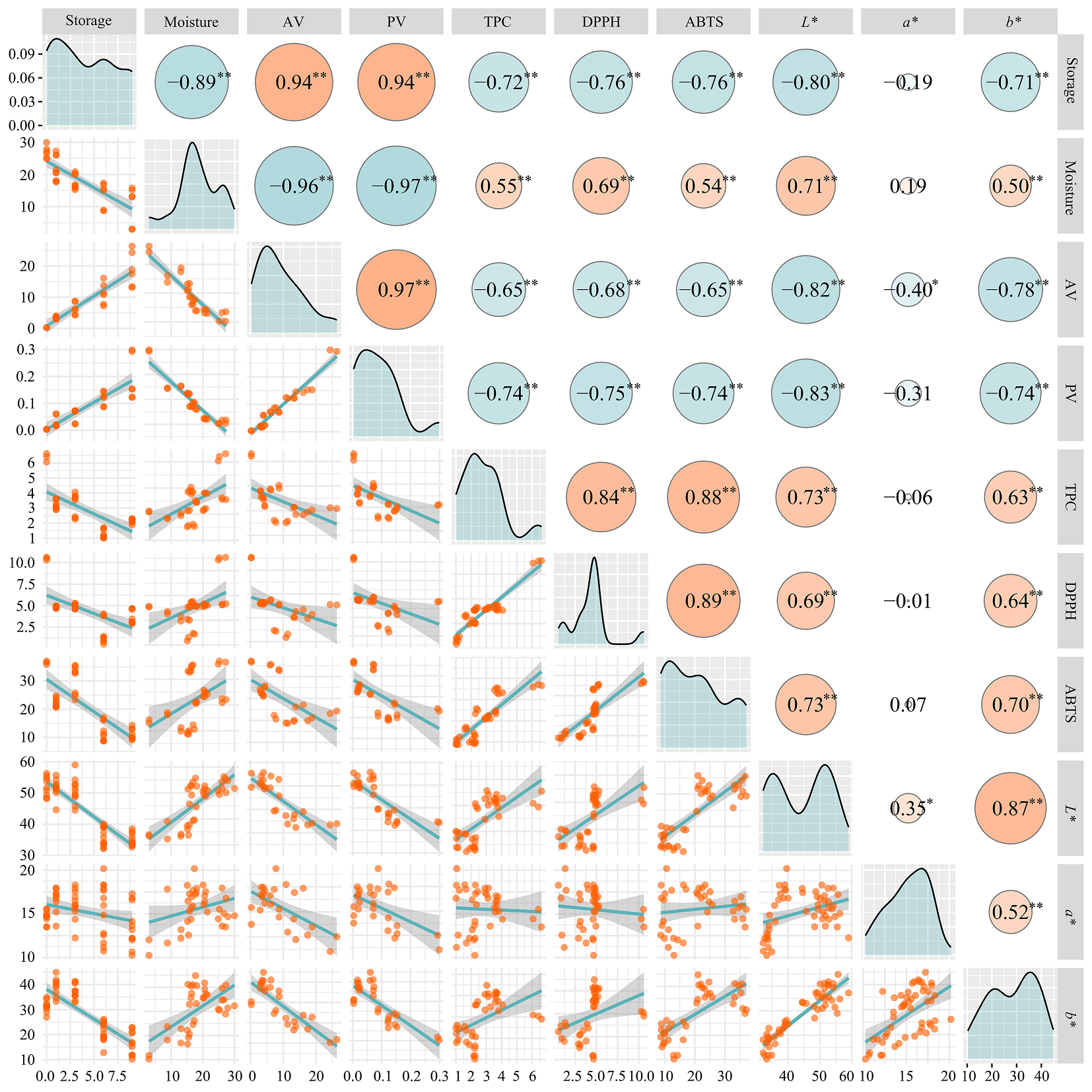

Supplement: Supplementary file 1 [file foods-14-00757-s001.zip › Figure S2.tif]

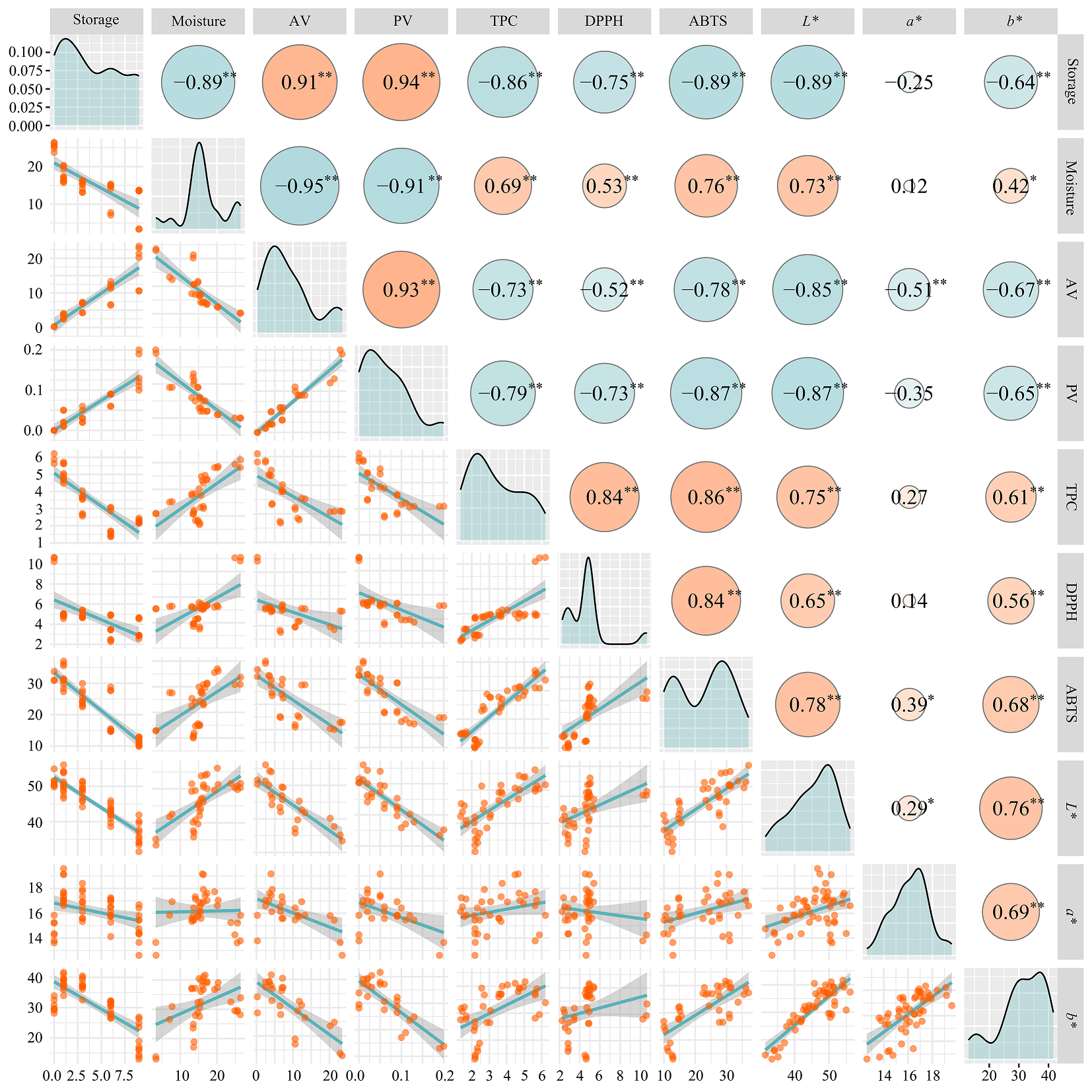

Supplement: Supplementary file 1 [file foods-14-00757-s001.zip › Figure S3.tif]

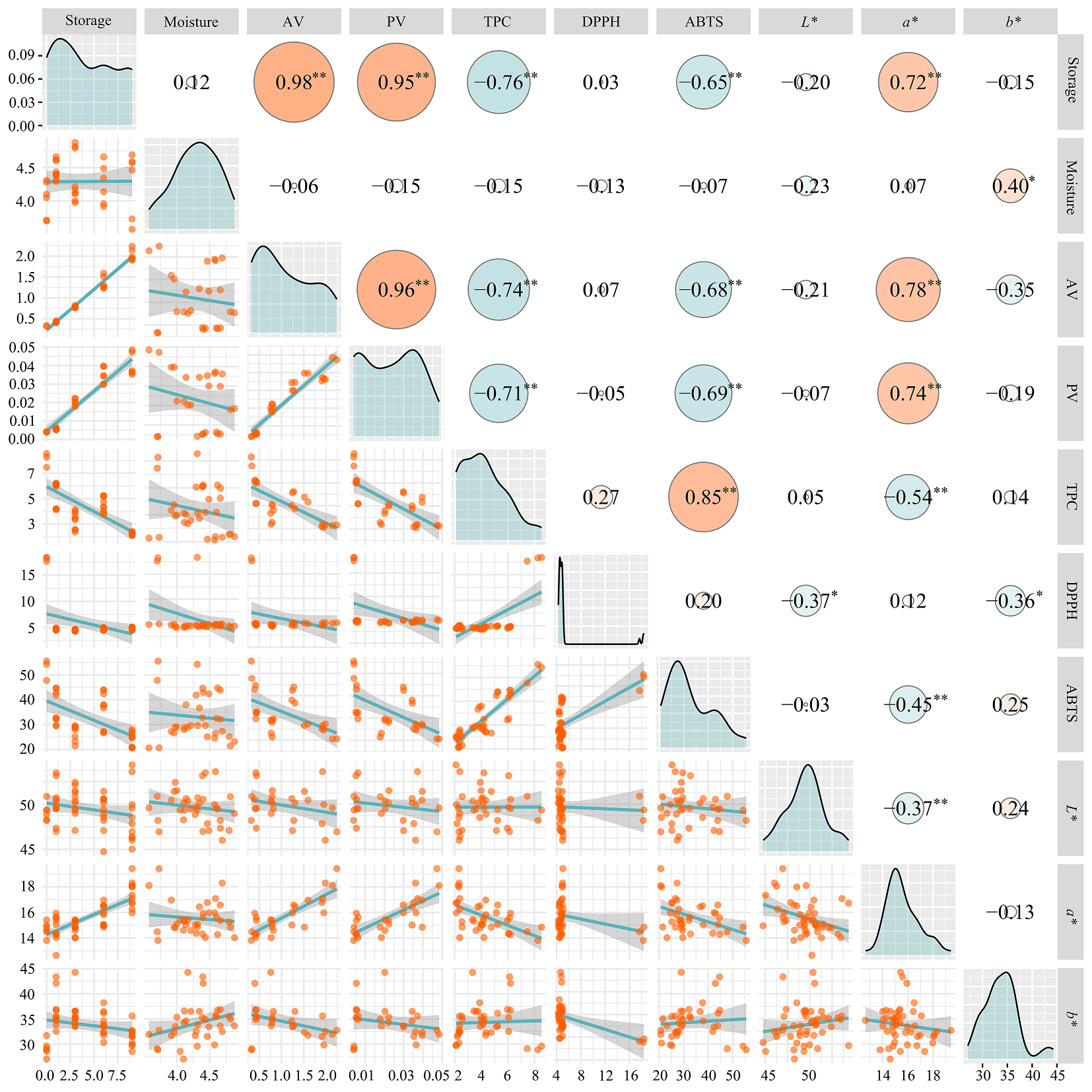

Supplement: Supplementary file 1 [file foods-14-00757-s001.zip › Figure S4.tif]
